# The characteristics of a good clinical teacher as perceived by resident physicians in Japan: a qualitative study

**DOI:** 10.1186/1472-6920-13-100

**Published:** 2013-07-25

**Authors:** Makoto Kikukawa, Hiromi Nabeta, Maiko Ono, Sei Emura, Yasutomo Oda, Shunzo Koizumi, Takanobu Sakemi

**Affiliations:** 1 Kyushu University, Japan Department of Medical Education, Kyushu University, 3-1-1 Maidashi Higashi-ku, Fukuoka 812-8285, Japan; 2 Department of Neuropsychiatry, Faculty of Medicine, Saga University, Saga, Japan; 3 Karatsu Municipal Hospital Kitahata, Saga, Japan; 4 Center for Graduate Medical Education Development and Research, Saga University Hospital, Saga, Japan; 5 Center for Comprehensive Community Medicine, Faculty of Medicine, Saga University, Saga, Japan; 6 Shichijo Clinic, Kyoto, Japan

**Keywords:** Resident, Clinical teacher, Culture, Qualitative study, Japan

## Abstract

**Background:**

It is not known whether the characteristics of a good clinical teacher as perceived by resident physicians are the same in Western countries as in non-Western countries including Japan. The objective of this study was to identify the characteristics of a good clinical teacher as perceived by resident physicians in Japan, a non-Western country, and to compare the results with those obtained in Western countries.

**Methods:**

Data for this qualitative research were collected using semi-structured focus group interviews. Focus group transcripts were independently analyzed and coded by three authors. Residents were recruited by maximum variation sampling until thematic saturation was achieved.

**Results:**

Twenty-three residents participated in five focus group interviews regarding the perceived characteristics of a good clinical teacher in Japan. The 197 descriptions of characteristics that were identified were grouped into 30 themes. The most commonly identified theme was “provided sufficient support”, followed by “presented residents with chances to think”, “provided feedback”, and “provided specific indications of areas needing improvement”. Using Sutkin’s main categories (teacher, physician, and human characteristics), 24 of the 30 themes were categorized as teacher characteristics, 6 as physician characteristics, and none as human characteristics.

**Conclusions:**

“Medical knowledge” of teachers was not identified as a concern of residents, and “clinical competence of teachers” was not emphasized, whereas these were the two most commonly recorded themes in Sutkin’s study. Our results suggest that Japanese and Western resident physicians place emphasis on different characteristics of their teachers. We speculate that such perceptions are influenced by educational systems, educational settings, and culture. Globalization of medical education is important, but it is also important to consider differences in educational systems, local settings, and culture when evaluating clinical teachers.

## Background

“In each culture, values are slightly different; people aspire after different aims, follow different impulses, yearn after a different form of happiness. In each culture, we find different institutions in which man pursues his life-interest, different customs by which he satisfies his aspirations, different codes of law and morality which reward his virtues or punish his defections.” (Malinowski, 1922. Argonauts of the Western Pacific [1]).

Globalization has definitely influenced medical practice [2], and medical education has now largely been globalized [3-5]. This trend has rapidly reformed Japanese medical education [6,7]. In 2004, Initial Postgraduate Clinical Training and the Matching System were started [8]. In 2006, the Japan Council for Evaluation of Postgraduate Clinical Training established Initial Postgraduate Clinical Training Quality Assurance, modeling the Accreditation Council for Graduate Medical Education [9]. In addition, all medical schools in Japan have introduced the Objective Structured Clinical Evaluation to assess clinical competency since 2006.

There is, however, growing evidence that perceptual processes are influenced by culture [10,11]. For example, Khine and Fisher (2004) reported that students perceived a more favorable interpersonal relationship with Western teachers than with Asian teachers in secondary school science classrooms. In medical education research, the question of how cultural differences affect medical education has attracted a great deal of attention, especially in the area of cultural competence [12-14].

Questions regarding the characteristics of good clinical teachers have been the subject of a considerable body of literature, ranging from essays to empirical studies. Discussion is ongoing, with the belief that better teaching should translate to better learning by physicians in training [15,16].

Sutkin et al. [16] reviewed 68 literature reports published between 1904 and 2006 regarding the characteristics of a good clinical teacher. They grouped descriptors into 49 themes that they clustered into three main categories: physician characteristics, teacher characteristics, and human characteristics (Table 1). They reported that 61 of these studies were performed in either the United States or Canada, and that little is known about the situation in other countries. There were no reports from Asian countries [16,17]. Therefore, it is not known whether the characteristics of a good clinical teacher are the same in Western countries as in non-Western countries.

**Table 1:** Sutkin's categories and themes (number of descriptions out of 480 in total)

**Characteristics**	**Themes**
Teacher characteristics	Maintains positive relationships with students and a supportive learning environment (27)
	Demonstrates enthusiasm for teaching (18)
	Is accessible/available to students (16)
	Provides effective explanations, answers to questions, and demonstrations (16)
	Provides feedback and formative assessment (15)
	Is organized and communicates objectives (14)
	Demonstrates knowledge of teaching skills, methods, principles, and their application (12)
	Stimulates students' interest in learning and/or subject (12)
	Stimulates or inspires trainees' thinking (11)
	Encourages trainees' active involvement in clinical work (11)
	Provides individual attention to students (10)
	Demonstrates commitment to improvement of teaching (10)
	Actively involves students (10)
	Demonstrates learner assessment/evaluation skills (7)
	Uses questioning skills (4)
	Stimulates trainees' reflective practice and assessment (4)
	Teaches professionalism (4)
	Is dynamic, enthusiastic, and engaging (3)
	Emphasizes observation (2)
	Other (5)
Physician characteristics	Demonstrates medical/clinical knowledge (30)
	Demonstrates clinical and technical skills/competence, clinical reasoning (28)
	Show enthusiasm for medicine (19)
	Models a close doctor-patient relationship (10)
	Exhibits professionalism (8)
	Is scholarly (6)
	Values teamwork and has collegial skills (6)
	Is experienced (3)
	Demonstrates skills in leadership and/or administration (2)
	Accepts uncertainty in medicine (1)
Human characteristics	Communication skills (21)
	Acts as role model-other (15)
	Is an enthusiastic person in general (14)
	Is personable (12)
	Is compassionate/empathetic (11)
	Respects others (11)
	Displays honesty/integrity (10)
	Has wisdom, intelligence, common sense, and good judgment (6)
	Appreciates culture and different cultural backgrounds (6)
	Considers others' perspectives, viewpoints (6)
	Is patient (4)
	Balances professional and personal life (4)
	Is perceived as a virtuous person and a globally good person (4)
	Maintains health, appearance, and hygiene (3)
	Is modest and humble (3)
	Has a good sense of humor (3)
	Is responsible and conscientious (3)
	Is imaginative (3)
	Has self-insight, self-knowledge, and is reflective (2)
	Is altruistic (2)
	Other (12)

The objective of this study was to identify the characteristics of a good clinical teacher as perceived by resident physicians in Japan, a non-Western country, and to discuss similarities and differences between the characteristics emphasized by Japanese residents and Sutkin’s study results.

## Methods

### Design

This study was designed with reference to the constant comparative method of the grounded theory approach [18]. Data collection was primarily by semi-structured focus group interviews (FGIs), which provide a qualitative method for collecting data, in which a moderator facilitates a group discussion of certain pre-defined issues [19]. This method allowed participants to interact with one another by questioning and challenging each other’s opinions, exchanging stories, and thinking out loud within a group of individuals who had similar experiences [20].

### Setting

This study was conducted in six residency programs (in university and community based hospitals) in Japan from February to June of 2009. After completion of a 6-year undergraduate program, Japanese residency programs focus on providing generalized training that will be useful regardless of the future specialty choices of the physicians. All residents rotate through three main specialties (more than 6 months of internal medicine, more than 3 months of emergency medicine, and more than 1 month of community-based medicine) during the first 2 years. Residents also rotate through at least two of surgery, anesthesiology, psychiatry, and obstetrics and gynecology, according to their own choices.

### Subject recruitment

The fourth author, a director of the Saga University Residency Program, was a key informant and recruited the participants. The participants were purposely sampled from the total of 123 residents in the six residency programs of Saga University Hospital (managed by university and community based hospitals).

Selected residents with at least 10 months of residency experience were invited to participate, because we expected these residents to have experienced many interactions with their clinical teachers. Residents were selected through maximum variation sampling that purposely selected participants by considering gender, specialties rotated through, and number of rotations. The selection process aimed to capture the perceptions of a broad range of residents regarding their supervision in a variety of clinical settings (university hospital, community hospital, and rural clinic).

The data saturation point was defined as the point at which new data no longer introduced additional themes [21].

### Participants and procedures

All participants gave written consent after reviewing written details of the study.

Data were collected and analyzed by three researchers (the first, second, and third authors). The first author is an experienced clinical teacher who received training in conducting FGIs from an experienced qualitative researcher, and was the primary interviewer. To minimize interview bias, the primary interviewer wrote down his thoughts about the topic beforehand and reflected on them so that he could accept participants’ opinions if they were different from his own [22]. The second author has a nursing background and was expected to have more neutral insights than the first author because she was not a medical doctor or a clinical teacher. The first and second authors were not involved in residency training. The third author is an experienced qualitative researcher.

All the FGIs were conducted in the quiet conference room of the Department of General Medicine. The first author facilitated all the FGIs according to the Interview Guide (Table 2), while the second author took notes. Questions focused on the characteristics of a good clinical teacher, with the more general and non-threatening questions being asked first. The first author ensured that every resident in the group participated. Soon after the FGIs finished, the first and second authors wrote extensive notes and discussed their impressions to capture main themes.

**Table 2:** Interview guidelines

	
1, Introductory conversation	Thanks, purpose of this study, informed consent, permission to record on audiotape.
2, Lead the interview using the following questions (note: care should be taken to respect the flow of discussion).	1. Please introduce yourselves.
	2. Think of good clinical teachers you have experienced, but do not identify them by name.
	3. Why do you feel they were good clinical teachers? Describe the teachers in detail regarding medical knowledge, skills, attitudes or behaviors, etc.
3, Conclusion	Would anybody like to say anything else about this topic?
Guidelines for the Interviewer	
1. Encourage participants to describe actual cases or situations.	
2. Discourage discussions of ‘ideal situations.’	

### Ethical approval

This research was approved by the institutional review board of Saga University Hospital.

### Data analysis

All the FGIs were audiotaped and transcribed verbatim immediately, using standard rules of transcription [20]. Identifiers and names were removed from the final transcripts. All transcripts were reviewed by the first author for accuracy. The first and second authors independently read and coded all transcripts, and then discussed, identified, and agreed on the coding of descriptors. The third author read all the transcripts independently, and discussed the identified themes with the first author. Finally, the first, second, and third authors agreed on the themes. This process of investigator triangulation was adopted to achieve high reliability of data analysis [23]. Through this process, the themes of a good clinical teacher as perceived by the focus groups were identified. The identified themes were grouped into the three main categories identified by Sutkin’s review of the literature (teacher, physician, and human characteristics), and were compared with those reported in Western countries.

## Results

A total of 23 residents agreed to participate in one of the five FGIs that were conducted. The characteristics of the participants are shown in Table 3. Each resident had experienced between 3 and 10 rotations, out of more than 13 possible rotations (Table 4). As no new themes were identified during the 5th focus group interview, thematic saturation was considered to have been reached.

**Table 3:** Descriptive characteristics of focus group participants

**Focus group**	**n**	**Male: Female**	**Mean age, years (range)**	**Mean months in training (range)**	**Number of rotations (range)**	**Specialty at the time of the focus group interview**
First	5	2:3	27 (25–35)	20.6 (11–23)	7 (3–10)	Pediatrics (2)
						Obstetrics and gynecology (1)
						Internal medicine (1)
						Emergency (1)
Second	4	2:2	34 (26–54)	23 (23)	9 (9–10)	Clinics (1)
						Psychiatry (1)
						Rehabilitation (1)
						Internal medicine (1)
Third	5	2:3	26 (26–27)	12 (12)	3 (3–4)	Clinics (3)
						Internal medicine (2)
Fourth	5	0:5	25 (25–26)	13 (13)	5 (4–5)	Clinics (3)
						Rehabilitation (1)
						Internal medicine (1)
Fifth	4	2:2	26 (25–27)	16.5 (14–24)	6 (5–9)	Clinics (3)
						Infectious diseases (1)
Overall	23	8:15	27 (25–54)	16.7 (11–24)	(3–10)	

**Table 4:** Specialties rotated through

**Specialty**	**Number of participants**
Internal medicine	23
Emergency	23
Surgery	20
Clinics	13
Psychiatry	13
Clinical laboratory	9
Pediatrics	8
Obstetrics and gynecology	8
Infectious diseases	8
Radiology	7
Anesthesiology	4
Rehabilitation	4
Palliative medicine	1

The 197 descriptions of characteristics of a good clinical teacher that were identified were grouped into 30 themes. The most commonly identified theme was “provided sufficient support”, followed by “presented residents with chances to think”, “provided feedback”, and “provided specific indications of areas needing improvement”

Using the three main categories of Sutkin’s review, 24 of the 30 themes were grouped as teacher characteristics, 6 as physician characteristics, and none as human characteristics (Table 5).

**Table 5:** Categorization of the 30 themes identified in the current study (number of descriptions out of 197 in total)

**Characteristics**	**Themes**
Teacher characteristics	Provided sufficient support (35)
	Presented residents with chances to think (24)
	Provided feedback (18)
	Provided specific indications of areas needing improvement (18)
	Was accessible (12)
	Provided residents with opportunities to practice (12)
	Established clear roles for residents (7)
	Did not criticize residents’ personalities (6)
	Treated individual residents equally (6)
	Provided opportunities for consultation (6)
	Did not get angry with residents (5)
	Was an enthusiastic teacher (4)
	Acknowledged residents as doctors (3)
	Actively involved residents in patient care (3)
	Provided clear explanations (3)
	Treated residents as equals (2)
	Thought about areas of uncertainty together with residents (2)
	Looked up information together with residents (2)
	Did not compare the ability of a resident with that of other residents (1)
	Was not boring (1)
	Established clear goals (1)
	Showed the next step (1)
	Provided references for further learning (1)
	Assessed residents (1)
Physician characteristics	Was enthusiastic about patient care (12)
	Did not get angry with patients (8)
	Demonstrated reasoning processes (6)
	Did not display bad manners (2)
	Established good relationships with medical staff (1)
	Did not pretend to know everything (1)
Human characteristics	None

### Teacher characteristics

#### ***Provided sufficient support (35 descriptions)***

Most residents wanted their teachers to provide a comfortable and safe learning environment, and appreciated it when their teachers offered support for effective learning. Quotations grouped in this theme include “When I cannot perform a procedure, a good teacher helps me”, “My teacher told me that he would complete the procedure if I was unable to do so, which allowed me to practice without excessive stress”, and “When I advised my patient of his condition, my teacher helped me, which allowed me to complete the consultation even though I was not confident.”

### Presented residents with chances to think (24)

Residents wanted to have chances to think during discussions with their teachers, and felt that such discussions were good learning opportunities. A typical quotation grouped in this theme is “My teacher discussed the management of my patient and asked me what drugs should be administered. I had a chance to think about it, which was a good learning opportunity.”

### Provided feedback (18)

Provision of both positive and negative feedback were considered to be important. Effective feedback led to increased motivation for patient care. Positive feedback encouraged residents’ confidence and constructive negative feedback was considered to be a good learning opportunity. Quotations grouped in this theme include “When I succeeded in taking a blood sample from my patient, my teacher praised me, which increased my confidence”, and “If I give a wrong answer, a good teacher points it out and explains it. I appreciate the feedback.”

### Provided specific indications of areas needing improvement (18)

Residents wanted specific indications of areas needing improvement from their teachers. This theme describes one aspect of feedback. As many residents considered that specific negative feedback was more effective that vague negative feedback, we included this as an independent theme. Quotations grouped in this theme include “My teacher gave specific indications of areas needing improvement, which was very helpful” and “I want specific negative feedback so that I know what needs to be improved.”

### Was accessible (12)

Residents wanted their teachers to be easily accessible for consultation, so that any issues could easily be discussed. Quotations grouped in this theme include “A good teacher was easily accessible, and allowed me to ask even silly questions” and “A good teacher allows me to ask any questions.”

### Provided residents with opportunities to practice (12)

Residents considered that effective teachers provided them with opportunities to practice. They wanted as many opportunities as possible, and found that these opportunities motivated them. Quotations grouped in this theme include “He gave me many chances to practice. I thought he was excellent” and “My best teacher provided me with chances to practice procedures, such as taking blood samples. I felt that I should make an effort to improve because of his consideration.”

### Established clear roles for residents (7)

Residents preferred to understand their role clearly. Conversely, they felt stress when their role was not clearly established. To reduce stress, they wanted their teachers to establish clear roles for residents. They considered that it was important to be acknowledged as a doctor. A typical quotation grouped in this theme is “My teacher clearly explains my tasks, so that I can do my job.”

### Did not criticize residents’ personalities (6)

Residents were afraid of being criticized for their personalities, which reduced motivation. They agreed that a good clinical teacher gives them negative feedback if necessary, but that this should never be a criticism of their personality. A typical quotation grouped in this theme is “A doctor told me that I was eccentric. I was so disappointed. I do not think he is a good teacher.”

### Treated individual residents equally (6)

Residents wanted to be treated equally by their teachers, and considered that a good teacher does not discriminate between residents. A typical quotation grouped in this theme is “My teacher treats individual resident equally, so I respect him.”

### Provided opportunities for consultation (6)

Residents regarded consultation of their teacher to be an important learning opportunity. They appreciated teachers who provided many opportunities for consultation, and found that this increased their satisfaction with learning. Quotations grouped in this theme include “My excellent teacher allows me to consult him at any time” and “One of my teachers provided me with many opportunities for consultation.”

### Did not get angry with residents (5)

Residents were afraid of being blamed for their actions by their teachers. They hesitated to consult teachers who might get angry, which reduced their learning opportunities and increased their stress levels. A typical quotation grouped in this theme is “A teacher blamed me for my performance in an angry voice in front of patients. A teacher who stays calm is good.”

### Was an enthusiastic teacher (4)

Residents considered that a good teacher should be enthusiastic about teaching. They felt that the enthusiasm of their teachers influence their motivation. A typical quotation grouped in this theme is “He took responsibility for my learning. He told me that my failure is his responsibility. He motivated me.”

### Acknowledged residents as doctors (3)

Residents wanted to be acknowledged as doctors by their teachers. They felt that this increased their confidence. A typical quotation grouped in this theme is “One of my teachers told me that I was a bad doctor and should resign. I have lost my confidence. A good teacher never says that you should stop being a doctor.”

### Actively involved residents in patient care (3)

Residents considered that a good teacher actively involved them in patient care, and that such involvement increased their motivation. Quotations grouped in this theme include “My teacher asks me about my patient assessments and management plans, and discusses them with me. I feel involved in patient care” and “He left me to do the task. I thought I should make every effort to complete it.”

### Provided clear explanations (3)

Residents considered that provision of clear explanations was important, and contributed to increased understanding. A typical quotation grouped in this theme is “My teacher explained the procedure to me, using a textbook. It was easy to understand and impressed me.”

### Treated residents as equals (2)

Residents considered that a good teacher considered their opinions when making decisions about patient care. Some residents were satisfied that they were treated as equals. A typical quotation grouped in this theme is “Even if my opinion is different from that of my teacher, he may consider my opinion. In a sense, I feel that he treats me as an equal.”

### Thought about areas of uncertainty together with residents (2)

Some residents preferred teachers who included them when thinking about areas of uncertainty, rather than teachers who had more medical knowledge. A typical quotation grouped in this theme is “One of my teachers said, “Let’s think through this problem together!” I was so grateful.”

### Looked up information together with residents (2)

Some residents learned how to look up information from their teachers, and felt that this was useful. A typical quotation grouped in this theme is “I asked my teacher a question, but he did not know the answer. He suggested that we look up the answer together. I learned how to find new information.”

### Did not compare the ability of a resident with that of other residents (1)

One resident expressed disappointment that a teacher compared him with another resident, resulting in a loss of confidence. “A doctor compared me with other residents in front of me. I was shocked. A good clinical teacher does not do that.”

### Was not boring (1)

One resident expressed appreciation of teachers who stimulated curiosity and interest. “A good teacher provides me with an interesting atmosphere, and does not bore me.”

### Established clear goals (1)

One resident wanted the goals of each rotation to be clearly discussed and identified by the teacher. He felt that this led to increased motivation. “I could discuss my goal for the rotation with my teacher. This helped me to maintain my motivation during the rotation.”

### Showed the next step (1)

One resident considered that it was useful for his teacher to indicate what to do next, when he was having trouble with something. “When I had trouble making a diagnosis, my teacher indicated what to do next.”

### Provided references for further learning (1)

One resident considered that it was useful for teachers to provide references for further learning. “My teacher recommended a good book regarding my patient’s operation, which was very helpful.”

### Assessed residents (1)

One resident expressed appreciation for teachers that assessed achievements, and felt that such assessment indicated good supervision. “He assessed my performance. I appreciate this, because it indicates that he supervised me properly.”

#### ***Physician characteristics***

##### Was enthusiastic about patient care (12)

Residents considered that their teachers’ enthusiasm for patient care provided a role model and influenced their motivation. Quotations grouped in this theme include “One of my teachers devoted himself to patient care. No matter how busy he was, he always saw his patients. I respect him. He is my role model” and “A doctor stayed until midnight to manage his patient. His effort motivated me.”

### Did not get angry with patients (8)

Residents considered that it was important for a good teacher to respect patients, and act as a role model. A typical quotation grouped in this theme is “I saw a doctor get angry with his patient. The patient became afraid of the doctor. One of my teachers respected his patients and never got angry with them.”

### Demonstrated reasoning processes (6)

Clinical reasoning was a topic of great interest to residents. Residents wanted to understand both the diagnosis and the reasoning process leading to the diagnosis. They considered it useful when teachers were able to explain this process. A typical quotation grouped in this theme is “It is useful when a teacher describes the reasoning process, because it can then be applied to other cases.”

### Did not display bad manners (2)

Some residents considered that a good teacher should have good manners to provide a good role model. A typical quotation grouped in this theme is “A doctor smoked in a place where smoking is not allowed. A teacher should not display such bad manners.”

### Established good relationships with medical staff (1)

One resident considered that a good teacher should be committed to working well within a multidisciplinary team. “One of my teachers tried to establish good relationships with medical staff in a new environment. I was motivated to do the same.”

### Did not pretend to know everything (1)

One resident considered that a good teacher would be honest about what they did and did not know. “I want a clinical teacher to tell me when he does not know the answers to my questions. That makes it easier for me to ask questions.”

## Discussion

Many qualitative and quantitative studies of characteristics of good clinical teachers have been reported in Western countries, but there are no such reports from Asian counties. This study aimed to identify the characteristics of a good clinical teacher as perceived by resident physicians in Japan, a non-Western country. The identified themes were compared with those reported in Western countries, according to the main categories identified by Sutkin’s review of the literature. Sutkin’s review identified 49 themes, including 19 teacher characteristics, 10 physician characteristics, and 20 human characteristics (Table 1). Using the same main categories, we categorized 24 of the 30 themes as teacher characteristics, 6 as physician characteristics, and none as human characteristics (Table 5).

We identified four main points of interest when comparing perceptions of the characteristics of a good clinical teacher between resident physicians in Japan and in Western countries.

First, most of the themes we identified were related to teacher characteristics, indicating that Japanese residents place importance on these characteristics. Paukert and Richards [24] reported that students’ descriptions of their teachers change from “teacher” to “supervisor” during their professional development. They reported that medical students valued the teacher role more than residents did. The Japanese Medical Practitioner Law (Ishi-hou) Article 17 states that no person is allowed to perform medical acts without a physician’s license [8]. Because of this restriction, undergraduate clinical education consists of observing instructors during medical interactions (bedside teaching) and practicing simulations of history taking and physical examinations with the consent of patients (bedside learning). Japanese medical students therefore tend to have less clinical training than students in Western countries [25]. We speculate that the high number of themes regarding teacher characteristics identified in our focus groups is a reflection of the observer role imposed on medical students in undergraduate medical education, and the fact that residents do not experience faculty members as supervisors. Japanese residents may experience a relatively low degree of interaction with their clinical teachers, and may therefore desire more interaction. Most of the themes frequently identified in this study were related to interactions with clinical teachers. Increased interaction seemed to motivate the residents. We speculate that physicians who have completed their resident programs and are enrolled in specialized fellowship programs may place more importance on physician and human characteristics. Further qualitative studies are needed to evaluate this possibility.

Second, “provided sufficient support” was the most commonly identified theme in our study. We considered this theme to correspond with “positive relationships with students and supportive learning environment” in Sutkin’s review. This theme therefore seems to be commonly identified both in Japan and in Western countries. This may be because this is a universal requirement for a good clinical teacher, regardless of the educational system, educational setting, and culture. It may also be that the high frequency with which this theme was identified in our study reflects insufficient clinical training of Japanese medical students because of the restrictions to undergraduate education described above. In addition, Japan has a severe shortage of doctors [26]. This limits the time available to instruct residents. Concerns about patient safety have also been rising rapidly in Japan [27]. Under these conditions, residents seem to depend on their relationships with faculty members to overcome anxiety [28]. In fact, support from clinical teachers has been identified as a factor reducing stress among residents in Japan [29]. Further studies are needed to investigate the reasons for this finding.

Third, surprisingly, medical knowledge was not identified as a theme by Japanese residents with a range of experience of residency programs and rotations, and clinical competence of teachers was not emphasized, even though these were the most commonly identified themes in Western countries. One resident said, “A clinical teacher who is always accessible surely has plenty of medical knowledge, because he always studies hard to teach us.” Another resident said, “I prefer a clinical teacher who is accessible, to one who has a lot of medical knowledge.” We therefore speculate that accessibility may be a more important characteristic than clinical competency. Another possibility is that the severe shortage of doctors in Japan increases the need for accessible teachers. This shortage may make it more difficult for Japanese residents to get medical knowledge from their teachers, and to learn from their teachers how to apply medical knowledge to patient management. Medical knowledge may not have been emphasized because residents can search for knowledge using internet resources such as PubMed and Google Scholar, but accessibility to teachers is important because residents have difficulty applying such information to patient management without assistance. Further studies are needed to evaluate the reasons for this finding.

Fourth, interestingly, themes related to human characteristics were not identified in this study. Students need different support from their teachers in East Asia including Japan, which has a background of Confucianism, compared with students in most Western countries [30,31]. In cultures influenced by Confucianism, high power distance is accentuated by communication behaviors [32]. Individuals from low power distance societies strive to reduce distance, and accentuate communication behaviors that support egalitarianism and closeness. Immediacy behaviors, which are defined as communication behaviors that reduce perceived distance between people, are in conflict with high power distance cultures and in congruence with low power distance cultures, as immediacy reduces physical and psychological distance through behaviors that convey interpersonal warmth, intimacy, and availability for communication [33]. Therefore, Japanese residents may not place importance on the human characteristics of their teachers. It is also possible that the relative lack of clinical training provided by the Japanese undergraduate medical education system causes residents to desire additional training, and therefore to place more importance on aspects of teaching and learning than on the personalities of their teachers. Further qualitative studies are needed to evaluate the reasons for this finding.

Considering our findings, we hypothesize that residents’ perceptions regarding a good clinical teacher can be influenced by the educational system, educational setting, and culture. Further investigation is needed to confirm this hypothesis.

### Limitations

We acknowledge several limitations of this study. First, it is possible for facilitators to influence opinions during FGIs. However, the potential for this is minimal in this study, because the facilitator was trained beforehand and conducted the FGIs according to carefully considered guidelines. Second, the data for this study were obtained from written transcripts of the FGIs, which is less accurate than directly obtaining data from audiotapes [34]. Third, our comparisons with Sutkin’s categories may differ from the comparisons of others. We consider our results to be significant in spite of these limitations, because this is the first study of its kind undertaken in Asia, and because the results appear to show some clear differences compared with the currently available literature in other parts of the world.

### Implications

The findings of this study indicate that residents’ perceptions of the characteristics of a good clinical teacher may not be universal, and may be influenced by the local educational system, educational setting, and culture. These differences in perception can affect which aspects of teachers should be evaluated. The methods used to evaluate teachers in Western countries may not be appropriate in Asian countries. Our findings show that medical educators should be aware of and sensitive to cultural differences, and should develop evaluation tools that are valid for their own cultures.

### Further research

Future research should focus on answering the following questions.

• Why do Japanese residents place so much emphasis on “teacher characteristics”? Does this change at different levels of training?

• Are residents’ responses influenced by their current specialty?

• Why was “provided sufficient support” so frequently identified as an important aspect of a good clinical teacher?

• Which aspect of teachers is more important: medical knowledge or accessibility? What are the reasons for this?

• Why did Japanese residents not emphasize the “human characteristics” of their teachers in this study?

• What are the perceptions of residents in other Japanese settings and other East Asian countries?

## Conclusions

In conclusion, we found that Japanese resident physicians emphasize teacher characteristics in their perceptions of characteristics of a good clinical teacher, but do not emphasize medical knowledge or clinical competence of the teacher, which are commonly emphasized in Western countries. This means that there are differences in the perceptions of residents between these regions, which can be influenced by the educational system, educational setting, and culture. An understanding of the characteristics of a good clinical teacher in each local educational context can help administrators to understand which aspects of clinical teachers should be evaluated. Medical education should be globalized, but educational systems, local settings, and cultural differences should also be considered when evaluating clinical teachers.

## Competing interests

The authors declare that they have no competing interests.

## Authors’ contributions

MK conducted the focus group interviews. MK, NH, and MO independently coded the data. ES recruited the participants. YO, SK, and TS all contributed substantially to the conception and design of the study, as well as to critical revisions of the paper. All authors approved the final manuscript.

## Pre-publication history

The pre-publication history for this paper can be accessed here:

http://www.biomedcentral.com/1472-6920/13/100/prepub
